# Development and evaluation of a modified brief assertiveness training for nurses in the workplace: a single-group feasibility study

**DOI:** 10.1186/s12912-017-0224-4

**Published:** 2017-06-06

**Authors:** Yohei Nakamura, Naoki Yoshinaga, Hiroki Tanoue, Sayaka Kato, Sayoko Nakamura, Keiko Aoishi, Yuko Shiraishi

**Affiliations:** 1 Department of Psychiatric and Mental Health Nursing, Graduate School of Nursing Science, University of Miyazaki, 5200 Kihara, Kiyotake, Miyazaki City, Miyazaki 889-1692 Japan; 2 Organization for Promotion of Tenure Track, University of Miyazaki, 5200 Kihara, Kiyotake, Miyazaki City, Miyazaki 889-1692 Japan; 3 Department of Psychiatric and Mental Health Nursing, School of Nursing, Faculty of Medicine, University of Miyazaki, 5200 Kihara, Kiyotake, Miyazaki City, Miyazaki 889-1692 Japan; 4 Center for Medical Education and Career Development, Faculty of Medicine, University of Miyazaki, 5200 Kihara, Kiyotake, Miyazaki City, Miyazaki 889-1692 Japan; 5 Department of Nursing, University of Miyazaki Hospital, 5200 Kihara, Kiyotake, Miyazaki City, Miyazaki 889-1692 Japan; 6 Faculty of Nursing, International University of Health and Welfare, 1-7-4 Momochihama, Sawara, Fukuoka City, Fukuoka 814-0001 Japan

**Keywords:** Assertiveness, Assertiveness training, Nurse, Education, Workplace

## Abstract

**Background:**

Effective communication has a great impact on nurses’ job satisfaction, team relationships, as well as patient care/safety. Previous studies have highlighted the various beneficial effects of enhancing communication through assertiveness training programs for nurses. However, most programs take a long time to implement; thus, briefer programs are urgently required for universal on-the-job-training in the workplace. The purpose of this feasibility study was to develop and evaluate a modified brief assertiveness training program (with cognitive techniques) for nurses in the workplace.

**Methods:**

This study was carried out as a single-group, open trial (pre-post comparison without a control group). Registered nurses and assistant nurses, working at two private psychiatric hospitals in Miyazaki Prefecture in Japan, were recruited. After enrolling in the study, participants received a program of two 90-min sessions with a 1-month interval between sessions. The primary outcome was the Rathus Assertiveness Schedule (RAS), with secondary measurements using the Brief Version of the Fear of Negative Evaluation Scale (BFNE) and the Brief Job Stress Questionnaire (BJSQ). Assessments were conducted at baseline and after a 1-month interval (pre- and post-intervention).

**Results:**

A total of 22 participants enrolled in the study and completed the program. The mean total score on the primary outcome (RAS) significantly improved from −12.9 (SD = 17.2) to −8.6 (SD = 18.6) (*p* = 0.01). The within-group effect size at the post-intervention was Cohen’s d = 0.24; this corresponds to the small effect of the program. Regarding secondary outcomes, there were no statistically significant effects on the BFNE or any of the BJSQ subscales (job-stressors, psychological distress, physical distress, worksite support, and satisfaction).

**Conclusions:**

This single-group feasibility study demonstrated that our modified brief assertiveness training for nurses seems feasible and may achieve a favorable outcome in improving their assertiveness. Further controlled trials with longer follow-up periods are required in order to address the limitations of this study.

**Electronic supplementary material:**

The online version of this article (doi:10.1186/s12912-017-0224-4) contains supplementary material, which is available to authorized users.

## Background

Effective communication has a great impact on nurses’ job satisfaction, team relationships, as well as patient care/safety [1]. Workplace communication issues among nurses are significantly related to burnout, reality shock, intention to leave, and less commitment to work [2–7]. Previous studies have also indicated that ineffective/poor communication in health care is a primary cause of health care errors. Nurses’ ability to be assertive is key in reducing risk, preventing major medical errors, and improving patient care because front-line nurses are well-positioned to observe early signs of unsafe conditions in care delivery [8–10]. Therefore, a positive work environment that fosters a culture of open communication among nurses is extremely important in attracting and retaining the best nursing staff, and in assuring quality patient care/safety.

Assertiveness enables nurses to enhance effective communication and to build effective team relationships because collaboration with other health care members needs both a high level of assertiveness and cooperation (i.e., meeting both one’s own and the other’s needs) [11]; however, for years nurses were said to lack assertiveness [1, 12]. Assertiveness is typically defined in terms of the legitimate and honest expression of one’s personal rights, opinions, feelings, beliefs, and interests without violating or denying the rights of others [13]. In the 1970s and 1980s, level of education was regarded as a key factor in nurses’ assertiveness [14, 15]. Although the number of nurses who have been educated at a higher academic level has increased from the 1990s, nurses still experience some difficulties asserting themselves even post-2000 [1, 12, 16]. Previous studies have suggested that nurses behave in a passive way, conforming to the stereotype of a ‘nice’ nurse, and are less likely to disagree with others [12, 16]. Cultural background (i.e. individualistic or collectivistic) and atmosphere of work environment also influence nurses’ assertive behavior [1, 17]. Thus, improving assertiveness skills among nurses is a key element of enhancing effective communication in health care and is very important in health care settings.

Assertiveness training programs have been developed to help the individual change how they view themselves, improve their assertiveness, and properly express their thoughts, feelings, and opinions [13]; however, most programs take a long time to implement. Typical full package training programs address the following topics: understanding the idea of assertive behavior, identifying an individual’s rights, classifying assertive behaviors, listening and asking questions, refusing unreasonable demands, making requests, responding to requests, appraising, expressing criticism, dealing with anger, and expressing approval and affection [13, 18]. Verbal or nonverbal communication skills are also employed (e.g. eye contact, body language, facial expressions, tone of voice, and the length of replies). Previous studies have highlighted the beneficial effects of assertiveness training on nurses such as improving assertiveness, reducing stress at work as well as depressive mood, preventing or lessening burnout, and improving self-esteem [19–21]. However, most studies employed programs that require a total time of over 10 h (e.g., 1.5 h × 10 sessions), and such a long program is difficult to implement as a universal on-the-job training in the workplace [22]. Thus, briefer training programs are urgently required.

A few studies have developed and evaluated the effectiveness of brief assertiveness training (total time < 5 h), but most of them had only minimal or no effects on participants’ assertiveness. To the best of our knowledge, four studies have employed a brief program for healthy participants (e.g. nurses, university students) using outcome measures for evaluating assertiveness (e.g., the Rathus Assertiveness Schedule) [6, 23–25]. When we calculated the pre-post effect sizes of these studies by the same formula (Cohen’s d = [M_pre_ − M_post_]/SD_pooled_) using raw data from each study, effect sizes ranged from −0.56 to 0.17 (i.e., there was no effect). However, it is possible that these brief programs could be improved. Two of these studies showed that there were no statistical significant effects on nurses’ assertiveness, but their brief programs were provided only through lectures [23, 24]. In order to promote developing assertiveness skills and to improve the transfer of acquired skills from training to real-life situations, some essential learning methods for adults have been identified, such as role-playing, modeling, feedback, and homework [13, 26–28]; thus, these practical learning methods should be employed even for short-term training. Further, all of these brief programs, as well as most typical full package programs, employed only behavioral techniques based on a skill-deficit model [28]. An alternate model of unassertive behavior assumes that the person has the requisite skills but is inhibited from behaving assertively by conditioned anxiety in assertive situations [29]. Timmins and McCabe [12] found that excessive fear of retribution or of others’ responses is a major barrier that prevents nurses from being assertive. In their survey, while many nurses were capable of acting assertively in certain situations, they held back for fear of upsetting the equilibrium of their workplace and/or interpersonal relationships. This is also an important point from a cultural perspective because three of the four studies that employed brief programs targeted Asian (Japanese) nurses [6, 23, 24]. In collectivistic Asian cultures, a high priority is placed on harmony within the group, respect for those with more authority (superiors), and the importance of saving face in social relations [17, 30, 31]. This has a considerable impact on Asian nurses’ communication styles [17]. Therefore, incorporating an approach to Japanese nurses’ perception/cognition relating to concerns about negative evaluation by others may improve a brief assertiveness training program. One study recruited 79 women (not Japanese nurses) and compared several specific methods as an assertiveness training: (1) behavioral rehearsal, (2) cognitive restructuring, (3) behavioral rehearsal and cognitive restructuring, (4) relationship control, and (5) wait-list control [32]. The results indicated that the combined behavioral rehearsal and cognitive restructuring was the most effective method in increasing assertive behavior and reducing emotional discomfort.

Thus, the purpose of this feasibility study was to develop and evaluate a modified brief assertiveness training (with cognitive techniques) for nurses in the hope that its shorter duration would increase its appeal to nurses in the workplace and its ease of implementation, facilitating subsequent dissemination. The main hypothesis is that our modified brief assertiveness training would increase participants’ assertiveness (primary outcome) and achieve a larger within-group effect size than those in previous studies that employed a brief program.

## Methods

### Design and setting

This feasibility study was carried out as a single-group, open trial from May to June 2016 at two private psychiatric hospitals in Miyazaki Prefecture, Japan. After enrolling in the study, the participants received a program consisting of two 90-min sessions (with cognitive techniques), with a 1-month interval between sessions. Assessments were conducted at baseline (pre-intervention) and 1-month (post-intervention) time points. For the baseline assessment, participants received questionnaires by mail (2 weeks before the first session), and mailed filled-out questionnaires to the study coordinator (by the day of the first session). For the 1-month assessment, participants received the same questionnaires after the second session, and mailed filled-out questionnaires to the study coordinator (within 2 weeks).

### Participants

Registered nurses and assistant nurses working at Wakakusa Hospital and Taniguchi Hospital were enrolled in the study. In Japan, there is a category of “assistant nurse”; while nursing practice does not differ greatly between registered nurses and assistant nurses, registered nurses have more education and a more advanced practice degree. Participants were recruited through posters placed at each hospital. The program was set as an on-the-job training (voluntary participation) at each hospital but novice nurses in the Wakakusa Hospital needed to attend the program as a required training.

### Modified brief assertiveness training program

The program was performed through lecture and role-playing in small groups of 3 participants. The program was provided by one leader and two facilitators from the University of Miyazaki (i.e. they were not nursing staff or managers from each study institution). The leader was the second author (N.Y.) who is a Japanese psychiatric nurse, and has experience of providing assertiveness training on more than two occasions. He also has over four years of experience in providing cognitive behavioral therapy (CBT) for mental disorders. The facilitators were two of the authors (Y.N. and H.T.). Both had received training in assertive skills, facilitation skills, role-play techniques, and group dynamics.

The overview of the program is shown in Table 1 (the original program text written in Japanese is presented as an Additional file 1). The program was developed based on existing assertiveness training literature [13, 33], and customized to be briefer by four of the authors (Y.N., N.Y., H.T., and Y.S.). The main training contents were basic knowledge about assertiveness (e.g., what assertiveness is, the right to assert oneself, and communication patterns) and practicing basic assertive behaviors (how to make requests, how to decline requests, and how to give and receive praise).

**Table 1:** Overview of the training program

Session	Theme	Content
1	Introduction	· Making groups (three members per group) · Self-introductions to get to know each other · Outline of the program and sharing the rules
	What is assertive	· Teaching what assertiveness is, the right to assert oneself, and communication patterns
	How to communicate assertively	· Teaching skills necessary for assertive communication
	Making requests	· Using assertive skills (making requests through role-play).
	Reviewing and setting an action plan	· Reviewing and summarizing the day’s session · Setting an action plan (homework)
2	Reviewing previous session	· Reviewing what we have learned in the previous session. · Sharing what I have done as homework with group members.
	Improving cognitive flexibility (restructuring dysfunctional thoughts)	· Using cognitive restructuring technique (Thought Challenging Record) to replace stress-inducing thought with more accurate, balanced, and less rigid thinking.
	Declining requests	· Using assertive skills (declining requests through role-play).
	Giving and receiving praise	· Using assertive skills (giving and receiving praise through role-play).
	Reviewing and setting action plan	· Reviewing and summarizing the day’s session · Setting an action plan

In order to shorten the program but maximize the training effect, the authors devised the following. First, the training program employed role-playing (in vivo) practice and was conducted in a group format. As mentioned earlier, practical learning methods are needed to promote the development of assertiveness skills and to improve the transfer of acquired skills from training to real-life situations [13, 26–28]. It is also believed that a group format is more beneficial than an individual format because members of the group can practice assertive behavior within the group and more easily accept assertive behaviors from other members [34, 35]. Second, we selected the (mentioned above) assertive behaviors in the program (how to make requests, how to decline requests, and how to give and receive praise) because many scholars regard these behaviors as a basic training level, and others are regarded as a more advanced level (e.g. how to handle criticisms, dealing with anger) [13, 36, 37]. Third, the program has typical scenarios for role-playing practice. In our experience, it takes a little time for some participants to remember and/or select certain difficult situations they faced in the past. For program development, the authors conducted a questionnaire survey for nurses at the research university hospital to understand their typical difficult situations. The results (214 valid responses received) revealed that nurses have three typical situations where they have difficulty in behaving assertively: communicating with senior or head nurses (*n* = 178, 83.2%), with patients or their family members (*n* = 112, 52.3%), and with other health care professionals (*n* = 82, 38.3%). This matches a past survey conducted in Ireland [12]. Based on this finding, the authors prepared three scenarios for each assertive behavior and participants selected one of them in the role-play. Fourth, there was a 1-month interval between sessions in order to build participants’ assertiveness skills through homework. Homework is important because participants are able to apply the skills they learned during sessions to multiple situations that arise in the workplace. In psychotherapy, clients who complete homework assignments have significantly better outcomes than clients who fail to do homework as part of their psychotherapy [38]. Therefore, participants were encouraged to practice assertive behaviors between sessions as homework using homework sheets prepared by the researchers (participants recorded their practice situation, how they acted assertively, results, what they have learned, etc.). Finally, we incorporated CBT techniques into the program [39, 40]. CBT is based on the principle that thoughts and perceptions can impact our feelings and behavior, and looks at ways to reassess negative thoughts so individuals can learn more flexible, positive ways of thinking that will subsequently influence their behaviors. A Thought Challenging Record (one of the cognitive restructuring techniques of CBT) [39] was applied to modify participants’ excessive fear of negative evaluation in order to promote assertive action before entering social situations.

### Outcomes

#### Rathus assertiveness schedule (primary outcome)

The primary outcome measure was self-reported degree of assertiveness, as measured on the Rathus Assertiveness Schedule (RAS) [41], which is the most frequently used scale for measuring assertiveness and the effects of assertiveness training [22]. The RAS consists of 30 items (including 16 inverted items) with a 7-point Likert scale scored from −3 (= very uncharacteristic of me) to 3 (= very characteristic of me). Total scores range from −90 to 90 points, with higher scores indicating a higher degree of assertiveness. Japanese versions of this questionnaire have been tested for reliability and validity [5, 42].

#### Brief version of the fear of negative evaluation scale (secondary outcome)

The Brief Version of the Fear of Negative Evaluation Scale (BFNE) comprises 12 items (including 4 inverted items) rated on a 5-point Likert-type scale ranging from 1 (= not at all characteristic of me) to 5 (= extremely characteristic of me) [43]. Total scores range from 12 to 60, with higher scores reflecting a higher concern for others’ evaluations. Good reliability and validity of the Japanese version have been reported [44].

#### Brief job stress questionnaire (secondary outcome)

The Brief Job Stress Questionnaire (BJSQ) is a 57-item multidimensional job stress questionnaire using 4-point Likert-type response options (1 = “strongly disagree” to 4 = “strongly agree”), to measure job-stressors (range 17–68), psychological distress (range 18–72), physical distress (range 11–44), worksite support (range 9–36), and satisfaction (range 2–8) [45]. Higher scores on job-stressors, psychological and physical distress indicate higher stressors or distress, and higher scores on worksite support and satisfaction indicate better worksite support and satisfaction. The validity and reliability of this questionnaire was previously examined [45].

### Analysis

The principle mode of analysis was paired *t*-tests to detect differences in the pre- and post-intervention scores of the outcome measures (RAS, BFNE, and BJSQ). The magnitude of the within-group effect was determined as the effect size based on Cohen’s d for each scale ([M_pre_ − M_post_]/SD_pooled_). Effect sizes are categorized as small (0.20–0.49), medium (0.50–0.79), and large (≥ 0.80). The analysis was conducted based on the intention-to-treat principle, and for non-completers, the last obtained data were carried forward. All statistical tests were two-tailed, and an alpha value of less than 0.05 was considered statistically significant. The statistical analysis was performed with IBM SPSS Statistics 22.0 for Windows (IBM Corp., Armonk, New York, USA).

Determining the optimal sample size for the study assures an adequate power to detect statistical significance, but we did not perform a sample size calculation because the current study was designed as a feasibility study.

## Results

### Baseline characteristics

**Fig. 1:**
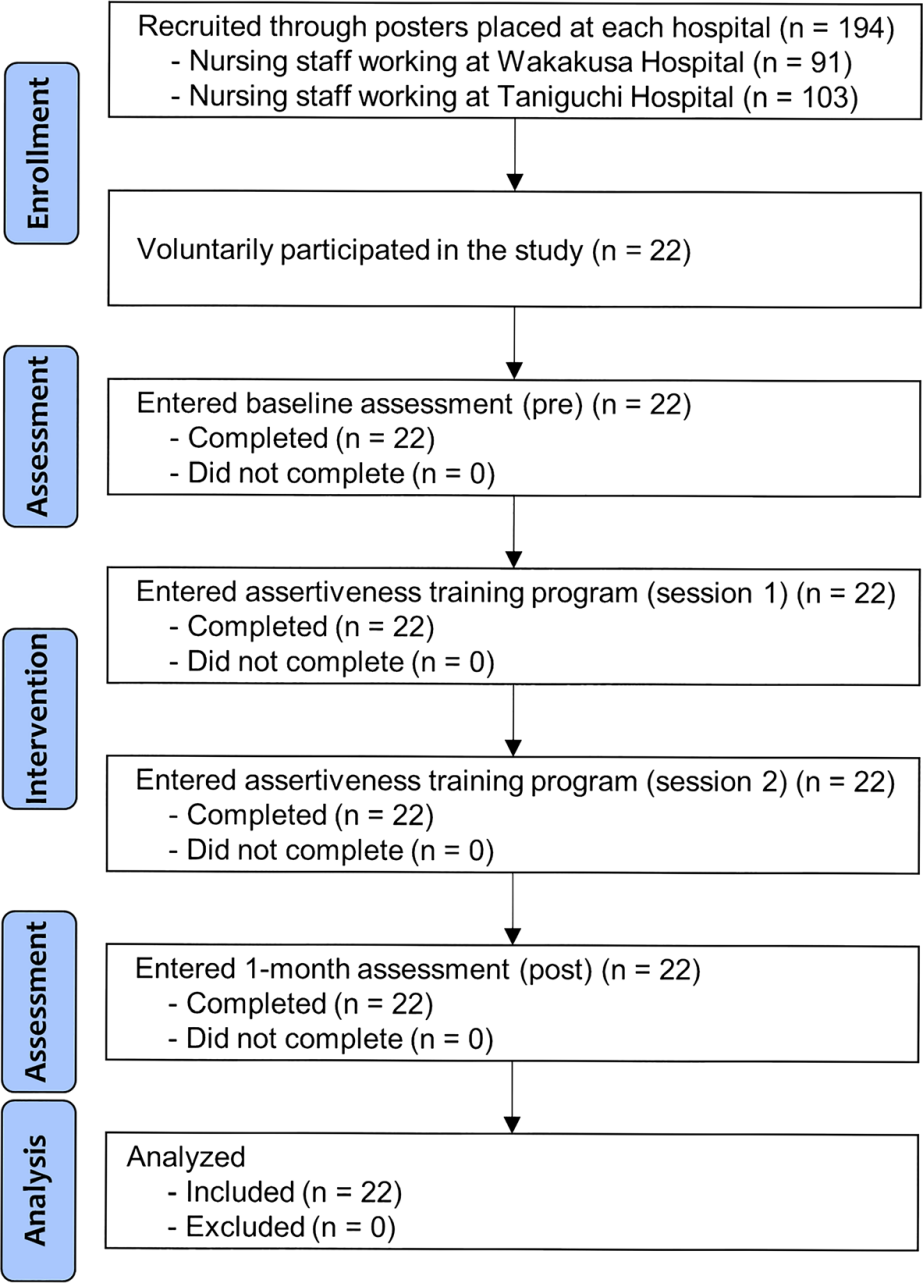
Participants’ flow diagram

The flow of participants through each stage is shown in Fig. 1. A total of 22 participants enrolled in this study and completed the program. The participants included 17 women (77.8%), and the mean age was 37.8 years (SD = 13.5). The average years of total work experience as a nurse was 13.5 (SD = 13.3), and work experience at one’s current workplace was 2.4 (SD = 3.9). Five novice nurses working at the Wakakusa Hospital (22.7%) attended the program as required training. Other demographic variables are presented in Table 2.

**Table 2:** Baseline characteristics of participants (*n* = 22)

Variable	Value
Gender, n (%)	
Male	5 (22.7)
Female	17 (77.3)
Age, years, mean (SD)	37.8 (13.5)
Certification, n (%)	
Registered nurse	18 (81.8)
Assistant nurse	4 (18.1)
Position, staff nurse, n (%)	13 (59.1)
Highest educational background	
High school	2 (9.1)
< 3 years of college/university	18 (81.8)
≥ 3 years of college/university	2 (9.1)
Work experience as a nurse, years, mean (SD)	
Total	13.5 (13.3)
Current workplace	2.4 (3.9)

### Outcomes

Table 3 shows the means and standard deviations of all measures at the baseline (pre) and 1-month (post) time points. With regards to the primary outcome, the mean total score on the RAS scores significantly improved from −12.9 (SD = 17.2) to −8.6 (SD = 18.6) over the study period (*p* = 0.01). The within-group effect size at the post-intervention was Cohen’s d = 0.24, which corresponds to the small effect of the program. As for secondary outcomes, there were no statistically significant effects on the BFNE (*p* = 1.00) or any of the BJSQ subscales (job-stressors, psychological distress, physical distress, worksite support, and satisfaction) (*p* = 0.28 to 0.79).

**Table 3:** Comparison of pre- and post-intervention scores of outcome measures

Measures	Baseline (Pre)		1-month (Post)		Difference		t	p	ES^‡^
	Mean	(SD)	Mean	(SD)	Mean	(SD)			
RAS (primary outcome)	−12.9	(17.2)	−8.6	(18.6)	−4.3	(7.4)	−2.7	0.01^*^	0.24
BFNE^**†**^	39.1	(7.0)	39.1	(6.8)	0.0	(4.1)	0.0	1.00	0.00
BJSQ									
Job-stressors^**†**^	39.6	(4.9)	40.2	(4.4)	−0.59	(2.8)	1.00	0.33	−0.13
Psychological distress^**†**^	37.4	(8.1)	37.1	(8.2)	0.32	(3.9)	0.38	0.71	0.04
Physical distress^**†**^	19.5	(7.0)	18.7	(5.9)	0.82	(3.4)	1.11	0.28	0.13
Worksite support	19.0	(3.8)	18.4	(4.7)	0.59	(2.6)	1.07	0.30	0.14
Satisfaction	4.5	(1.2)	4.4	(1.3)	0.45	(0.79)	0.27	0.79	0.04

^*^ *p* < 0.05, ^**^ *p* < 0.01, ^**†**^higher scores indicate greater severity or distress. ^**‡**^ categorize as small (0.20–0.49), medium (0.50–0.79), and large (≥ 0.80). Abbreviation: RAS, Rathus Assertiveness Schedule; BFNE, Brief Version of the Fear of Negative Evaluation Scale; BJSQ, Brief Job Stress Questionnaire; ES, Effect Size

## Discussion

This single-group feasibility study demonstrated that modified brief assertiveness training could lead to a significant improvement in assertiveness among psychiatric nurses. In particular, our pre-post effect size of 0.24 (small effect) in terms of the RAS was larger than the effect sizes ranging from −0.56 to 0.17 (indicating no effect) reported in previous studies that employed a brief program (total time < 5 h) [6, 23–25]. Furthermore, the mean total score of the RAS increased from −12.9 to −8.6, which corresponds to clinical improvement from outside of the optimal range (< −10) to inside of the optimal range (≥ −10). According to a longitudinal study of novice nurses [4], a graph of the incidence of burnout corresponding to RAS score exhibited a U-shape curve with the bottom scores between −10 and 10.

Although it is difficult to explain why our program achieved such a favorable outcome, the following reasons may account for this. First, most participants participated voluntarily in this study, so they may have had high preferences, motivation, and allegiance to the program. Second, we applied cognitive techniques in the program in order to modify nurses’ fear of others’ negative responses. However, it is impossible to determine the specific effect of applying cognitive techniques because there was no controlled comparison in this study. Finally, the program was led by only one nurse who had sufficient training and experience in assertiveness training and group therapy before the study. These factors might have contributed to a larger intervention effect in the study. However, our finding in this feasibility study will contribute in estimating the effect size of modified assertive training on nurses’ assertiveness, and in determining the optimal sample size for future studies in order to have a high probability (power) of detecting as statistically significant the clinically important difference of given sizes, if such a difference exists.

There are some questionnaire and intervention studies that measured the degree of assertiveness (RAS) among Japanese nurses, but most of them targeted only novice nurses or nurse managers [5, 44, 46–48]. These studies indicated that average RAS scores among nurse managers (range: −6.7 to −2.0) are higher than those among novice nurses (−12.4 to −12.1); thus, baseline RAS scores of −12.9 in this study population are closer to those of novice nurses. In addition to a paucity of comparable Japanese literature, the current study had a small sample and recruited nurses from psychiatric hospitals; thus, it is difficult to evaluate the generalizability and reliability of our study sample. However, the current study did not target a specific subgroup of nurses (i.e. novice nurses, mid-career nurses, nurse managers participated), so this is a strong point and the findings are informative not only for novice nurses or nurse managers but also for mid-career nurses.

Notably, individuals from collectivistic Asian cultures have greater levels of social anxiety and more fear of negative evaluation than those from individualistic cultures [30, 31, 49]. Participants in this study reported greater levels of baseline BFNE scores of 39.1 than Irish nurses (range: 32.7 to 34.7), but comparable to Japanese undergraduate students (range: 38.4 to 39.8) (no data for Japanese nurses) [50–53]. The assertive training program in this study incorporated cognitive restructuring techniques in order to modify nurses’ fear of negative evaluation. As introduced in the background, Linehan et al. [32] successfully demonstrated the effectiveness of assertiveness training that combined behavioral rehearsal and cognitive restructuring, but the training was implemented through 12 sessions. For further modification of our developed training program, extending each training session (e.g. from 90 min to 120 min) or adding one more session (e.g. from 2 sessions to 3 sessions) is worth considering so that participants are able to work more on cognitive restructuring.

There was no significant impact on job-related stress (BJSQ) throughout the study; there were several conflicting results for this in previous studies. For example, one study supports our finding that the 3-week assertiveness training for hospital nurses improved assertion knowledge and voluntary behavior, but had no impact on job-related stress until the end of the 1-month follow-up assessment [54]. On the other hand, another study revealed that intensive assertiveness training achieved significant improvement in both assertiveness and occupational stress among nurses [55]. More detailed investigation with larger samples is needed to evaluate the various effects of brief assertiveness training for nurses. Such investigation would include performing subgroup analysis (e.g. high- and low-assertiveness groups, current position, study institution, etc.) and/or only targeting high-risk nurses, and/or conducting longer follow-up observation (e.g. 1-year follow-up).

We must be cautious about the interpretation of the results, because the present study contains the following limitations. First, our study had a relatively small number of participants, resulting in limited generalizability of its conclusions. Second, this study targeted only psychiatric nurses; future studies should replicate our findings in different populations (e.g., recruited nurses from general hospital, nurse manager, Clinical Nurse Specialists, and Nurse Practitioner). Third, this was a single-group study without a control group; we could not conclude definitively that our program was effective (i.e., could not determine an intervention-specific effect). Fourth, the lack of follow-up data limits the generalizability of the study’s conclusions to longer-term outcomes. Fifth, the program was delivered by only one experienced nurse; thus, the high quality of the program leader might contribute to larger effects.

## Conclusions

Despite several limitations, our modified brief assertiveness training program—which comprised two 90-min sessions (total 180 mins) with cognitive techniques—seems feasible and may achieve favorable outcomes for nurses in improving their assertiveness. Further controlled trials with longer follow-up periods are required in order to address the limitations of this study.

## Additional file

Additional file 1: Original program text (in Japanese). (PDF 2880 kb)

## Abbreviations

BFNE: Brief version of the Fear of Negative Evaluation Scale
BJSQ: Brief Job Stress Questionnaire
RAS: Rathus Assertiveness Schedule

## References

[1] Garon M (2012). Speaking up, being heard: registered nurses’ perceptions of workplace communication. J Nurs Manag.

[2] Azuma T, Suzuki E (2007). Factors affecting occupational commitment among novice nurses in university hospitals. J Jpn Acad Nurs Adm Policies..

[3] Itomine I, Suzuki E, Kanoya Y, Sato C (2006). Factors related to reality shock experienced by new graduate nurses at university hospitals. J Jpn Soc of Nurs Res..

[4] Suzuki E, Kanoya Y, Katsuki T, Sato C (2006). Assertiveness affecting burnout of novice nurses at university hospitals. Jpn J Nurs Sci.

[5] Suzuki E, Kanoya Y, Kitaoka-Higashiguchi K, Sato C (2005). Workplace environment, assertiveness and burnout risk among novice nurses in university hospitals. J Jpn Soc of Nurs Res.

[6] Suzuki E, Saito M, Tagaya A, Mihara R, Maruyama A, Azuma T (2009). Relationship between assertiveness and burnout among nurse managers. Jpn J Nurs Sci.

[7] Spence Laschinger HK, Leiter M, Day A, Gilin D (2009). Workplace empowerment, incivility, and burnout: impact on staff nurse recruitment and retention outcomes. J Nurs Manag.

[8] Manning ML (2006). Improving clinical communication through structured conversation. Nurs Econ.

[9] Benjamin DM (2003). Reducing medication errors and increasing patient safety: case studies in clinical pharmacology. J Clin Pharmacol.

[10] McVanel S, Morris B (2010). Staff's perceptions of voluntary assertiveness skills training. J Nurses Staff Dev.

[11] Boone BN, King ML, Gresham LS, Wahl P, Suh E (2008). Conflict management training and nurse-physician collaborative behaviors. J Nurses Staff Dev.

[12] Timmins F, McCabe C (2005). Nurses’ and midwives’ assertive behaviour in the workplace. J Adv Nurs.

[13] Alberti RE, Emmons ML (2008). Your perfect right: assertiveness and equality in your life and relationships.

[14] Gerry EM (1989). An investigation into the assertive behaviour of trained nurses in general hospital settings. J Adv Nurs.

[15] Kilkus SP (1993). Assertiveness among professional nurses. J Adv Nurs.

[16] Canam CJ (2008). The link between nursing discourses and nurses’ silence: implications for a knowledge-based discourse for nursing practice. ANS Adv Nurs Sci.

[17] Xu Y, Davidhizar R (2005). Intercultural communication in nursing education: when Asian students and American faculty converge. J Nurs Educ.

[18] Lin YR, Wu MH, Yang CI, Chen TH, Hsu CC, Chang YC (2008). Evaluation of assertiveness training for psychiatric patients. J Clin Nurs.

[19] Shimizu T, Kubota S, Mishima N, Nagata S (2004). Relationship between self-esteem and assertiveness training among Japanese hospital nurses. J Occup Health.

[20] Katsuhara Y, Mashino S (2001). Development and evaluation of an assertiveness training program for Japanese nurses. Coll Nur Art Sci Hyogo Bull.

[21] Shimizu T, Mizoue T, Kubota S, Mishima N, Nagata S (2003). Relationship between burnout and communication skill training among Japanese hospital nurses: a pilot study. J Occup Health.

[22] Tateishi A (2012). A literature review about the effects of assertion training on healthy people. J Health Care Nurs.

[23] Honjo T, Komada R (2015). Effectiveness of psychoeducation for health care professionals: asssertive training. Proc Jpn Acad Ophthal Nurs.

[24] Yamamoto K, Ishida S, Shimizu K, Hanada F (2015). The interventional effect of the short form of assertion training for preceptor nurses. J Jpn Health Med Assoc.

[25] Zielinski JJ, Williams LJ (1979). Covert modeling vs. behavior rehearsal in the training and generalization of assertive behaviors: a crossover design. J Clin Psychol.

[26] Hersen M, Eisler RM, Miller PM, Johnson MB, Pinkston SG (1973). Effects of practice, instructions, and modeling on components of assertive behavior. Behav Res Ther.

[27] McFall RM, Marston AR (1970). An experimental investigation of behavior rehearsal in assertive training. J Abnorm Psychol.

[28] McFall RM, Twentyman CT (1973). Four experiments on the relative contributions of rehearsal, modeling, and coaching to assertion training. J Abnorm Psychol.

[29] Wolpe J (1982). The practice of behavior therapy.

[30] Gao G (1998). “Don't take my word for it.”—understanding Chinese speaking practices. Int J Intercult Relat.

[31] Schreier SS, Heinrichs N, Alden L, Rapee RM, Hofmann SG, Chen J (2010). Social anxiety and social norms in individualistic and collectivistic countries. Depress Anxiety.

[32] Linehan MM, Goldfried MR, Goldfried AP (1979). Assertion therapy: skill training or cognitive restructuring. Behav Ther.

[33] Hiraki N (2009). Assertion training: for smooth self-expression.

[34] Lange AJ, Jakubowski P, McGovern TV (1976). Responsible assertive behavior: cognitive/behavioral procedures for trainers.

[35] Lin YR, Shiah IS, Chang YC, Lai TJ, Wang KY, Chou KR (2004). Evaluation of an assertiveness training program on nursing and medical students’ assertiveness, self-esteem, and interpersonal communication satisfaction. Nurse Educ Today.

[36] Aschen SR (1997). Assertion training therapy in psychiatric milieus. Arch Psychiatr Nurs.

[37] Kirkpatrick H, Forchuk C (1992). Assertiveness training: does it make a difference?. J Nurs Staff Dev.

[38] Kazantzis N, Whittington C, Zelencich L, Kyrios M, Norton PJ, Hofmann SG (2016). Quantity and quality of homework compliance: a meta-analysis of relations with outcome in cognitive behavior therapy. Behav Ther.

[39] Beck AT, Rush AJ, Shaw BF, Emery G (1979). Cognitive therapy of depression.

[40] Yoshinaga N, Matsuki S, Niitsu T, Sato Y, Tanaka M, Ibuki H (2016). Cognitive behavioral therapy for patients with social anxiety disorder who remain symptomatic following antidepressant treatment: a randomized, assessor-blinded, controlled trial. Psychother Psychosom.

[41] Rathus SA (1973). A 30-item schedule for assessing assertive behavior. Behav Ther.

[42] Suzuki E, Kanoya Y, Ishida S, Katsuki T, Sato C (2004). The development of the Japanese version of the Rathus assertiveness schedule. Jpn J Human Sciences Health Soc Serv.

[43] Leary MR (1983). A brief version of the fear of negative evaluation scale. Personal Soc Psychol Bull.

[44] Maruyama A, Suzuki E (2004). Characteristics of novice nurses in pediatric wards at university hospitals. J Jpn Acad Nurs Adm Policies.

[45] Shimomitsu T, Yokoyama K, Ono Y, Maruta T, Tanigawa T, Kato M (2000). The final development of the brief job stress questionnaire mainly used for assessment of the individuals. Ministry of Labour sponsored grant for the prevention of work-related illness.

[46] Suzuki E, Kanoya Y, Katsuki T, Sato C (2007). Verification of reliability and validity of a Japanese version of the Rathus assertiveness schedule. J Nurs Manag.

[47] Suzuki E, Saitho M, Maruyama A, Azuma T, Katsuki T, Sato C (2007). Verification of reliability and validity of the Japanese version of the Rathus assertiveness schedule (J-RAS) among executive nurses. Jpn J Human Sci Health-Soc Serv.

[48] Suzuki E, Tagaya A, Matsuura R, Saito M, Maruyama A, Azuma T (2009). Comparison of burnout scores before and after assertiveness training among nurse managers. The J Jpn Acad Nurs Adm Policies.

[49] Heinrichs N, Rapee RM, Alden LA, Bogels S, Hofmann SG, Oh KJ (2006). Cultural differences in perceived social norms and social anxiety. Behav Res Ther.

[50] Morimoto S, Tanno Y (2004). A study of paranoid ideation in college students: an approach with the diathesis-stress model:an approach with the diathesis-stress model. Jpn J Psychol.

[51] Begley CM, White P (2003). Irish nursing students’ changing self-esteem and fear of negative evaluation during their preregistration programme. J Adv Nurs.

[52] Sasagawa S, Kanai Y, Muranaka Y, Suzuki S, Shimada H, Sakano Y (2004). Development of a short fear of negative evaluation scale for Japanese using item response theory. Jpn J Behav Ther.

[53] Shirotsuki K, Sasagawa S, Nomura S (2009). The effect of speech estimation on social anxiety. Jpn J Psychol.

[54] Yamagishi M, Kobayashi T, Kobayashi T, Nagami M, Shimazu A, Kageyama T (2007). Effect of web-based assertion training for stress management of Japanese nurses. J Nurs Manag.

[55] Lee S, Crockett MS (1994). Effect of assertiveness training on levels of stress and assertiveness experienced by nurses in Taiwan, republic of China. Issues Ment Health Nurs.

