# Supplementary material for: Development and evaluation of a modified brief assertiveness training for nurses in the workplace: a single-group feasibility study
Source: BMC Nurs. 2017 Jun 6;16:29. doi: 10.1186/s12912-017-0224-4 (PMC5461750; doi:10.1186/s12912-017-0224-4)
Supplement: Additional file 1: — Original program text (in Japanese). (PDF 2880 kb) [file 12912_2017_224_MOESM1_ESM.pdf]

For Nurses

Assertiveness Training

-Communication style that is respectful of others but clear and firm in intent-

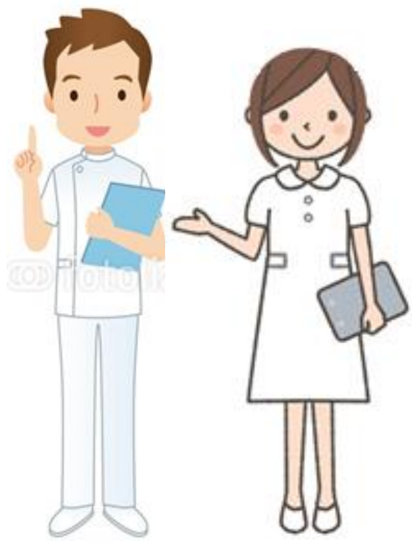

University of Miyazaki, Japan

看護職向け

アサーティブ・トレーニング

～相手も自分も尊重したコミュニケーション～

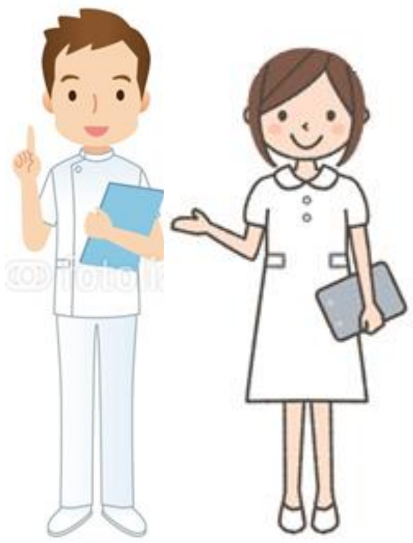

宮崎大学

目次

＜第１部＞

1. アサーティブとは ・・・・・・3

2. アサーティブな
コミュニケーションのコツ ・・・・・・10

3. 上手に頼む
（ロールプレイ①） ・・・・・・14

＜第2部＞

4. 柔軟な考え方を身につけよう ・・・・・21

5. 上手に断る
（ロールプレイ②） ・・・・・31

6. 上手にほめる・ほめられる
（ロールプレイ③） ・・・・・36

アサーティブとは…

医療現場ではどのような人たちとのコミュニケーションが必要でしょうか？

看護師

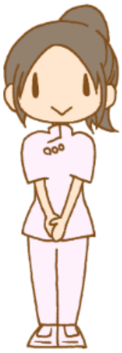

患者とその家族

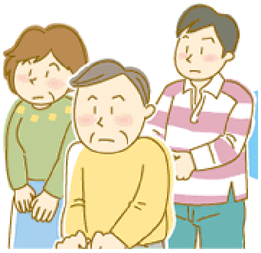

・適切な信頼関係
・治療方針への相互理解
・情報収集
・治療、ケア、指導等

看護師同士

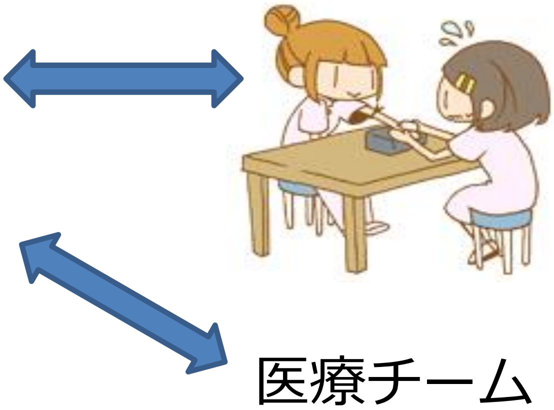

・同僚、上司、後輩との関係
・情報共有（報告、連絡、相談）

医療チーム

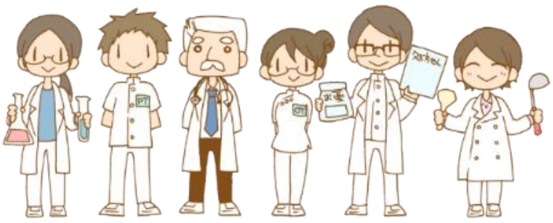

・他職種との関係
・情報共有
・調整役

自分の気持ちや考えを率直に上手に伝えることを難しいと感じることはありませんか？

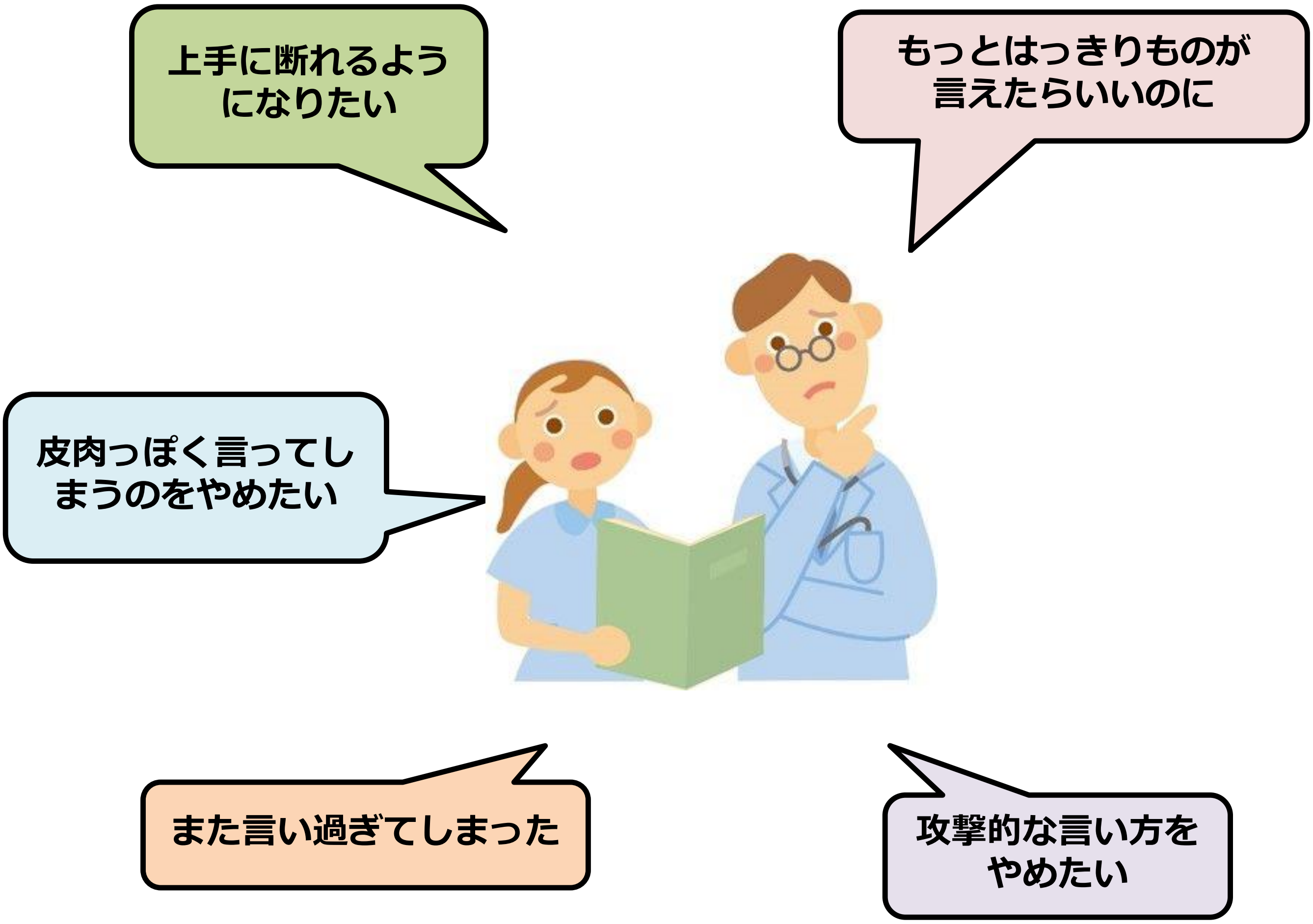

どうやったら自分の気持ちや考えを率直に上手に伝えて心地よいコミュニケーションを行うことができるようになるのでしょうか？

アサーティブ

「相手を尊重しつつ自分の権利も尊重し、誠実に、率直に、対等に自己表現すること」

自分も相手も大切にして

伝えたいこと
伝えなければならないこと
} 内容

を伝わるように話す

「どう伝えて」「どう受け取ってもらうか」が重要であり、その結果、**相手がどのように反応するかはアサーティブに含まれない**。

アサーティブである「権利」

自分の意見や感情を表現すること・・・

→ 私たち誰もが持つ**「基本的な権利」**

アサーティブなコミュニケーションは、

「全ての人に自己表現の権利があり、自己表現の権利を自分にも相手にも同じように認める」ことから始まる。

アサーティブである「権利」

- **感じたこと・考えたことを表現して良い**
  - ・自分の感情と意見をもち、それを表明する権利
  - ・欲しいものを望む権利
  - ・「Yes」「No」と言う権利
- **人間は不完全である**
  - ・失敗する権利・間違う権利とそれに責任を持つ権利
  - ・「わからない」「知らない」と言う権利
- **人は違っていて良い**
  - ・自分の話しに耳を傾けてもらう権利
  - ・自分の時間や身体、所有物をどうするか決める権利
  - ・自分の価値観を大切にする権利
  - ・尊重され、面目を保つ権利
- **アサーティブでいなくても良い**
  - ・自分の意見を主張しないでいる権利

アサーティブが低いと…

●日常生活では

・言いたいことが言えず、ストレスがたまる
・誤解されたり、伝わらなくて苛立ちがつのる
・人とコミュニケーションを取ることに消極的になる
・頭ごなしに怒って雰囲気が悪くなる

●医療現場では

・情報の誤認や未伝達からインシデントや医療事故に発展する
・チーム医療の不活化
・医療職者の対人ストレスが増加し、患者さんへの悪影響につながる
・バーンアウト（燃え尽き）の危険因子となる

※言いたいことが言えない場合（＝自分の権利を尊重できていない）だけでなく、自分の言いたいことを頭ごなしに相手に言ってしまう場合（＝相手の権利を尊重していない）も、アサーティブが低いということになります。

コミュニケーションの３つのモード

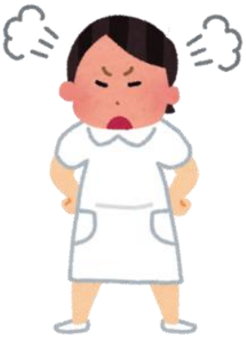

いかりモード

攻撃的で人に食ってかかる

・人間関係を勝ち負けで判断
・相手を見下し、自分が優位であると示す
・言動が相手を傷つけることが多い
・周囲はビクビク

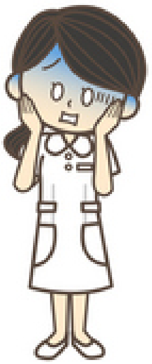

ひるみモード

自己犠牲的でふみにじられても我慢する

・自分が我慢すれば良いと感じている
・自分で選択・決定しようとしない
・自分を常に卑下している
・人と対立することを極力避け、責任逃れする

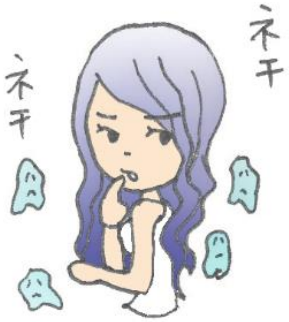

いやみモード

攻撃性を隠して相手をコントロールする

・表立ったことを荒立てたりしないで仕返しをする
・周囲の人間を利用し相手に罪の意識を持たせる
・暗に嫌味やけなしを言う
・周りは傷つくが、証拠がなく責められない

モードはその時の状況や場面によって変わります
大切なことは「自分がいま、どのモードなのか」を知ることです

看護師はなぜアサーティブになりにくい？

- 人の役に立ちたい気持ちの強さ
  （自分を抑えて相手を優先）
- 常に共感的で優しい看護師であるべき
- 我慢が高じて攻撃的になってしまうことも
- チームで仕事をする上で、医師に従順になりやすい
- 多忙・不規則な勤務形態で自分にも余裕がない

こんなときどうしますか？

<u>患者の食事介助中に医師から要件を頼まれた看護師Aさん</u>

看護師Aさんが配膳をしていると、ここ数日食欲がなくほとんど食事を食べていなかった患者Bさんが「今なら食べられそう」と言う。看護師Aさんは「食欲を感じているこの機会を逃したくない」と思い、急いで配膳を済ませて食事介助を始めた。
すると、突然部屋に医師のCさんがやってきて「（他の患者さんの）包帯交換をするからすぐに来て」と言われた。
医師のCさんは手術件数が多く忙しいため、昼休みの時間を使って包帯交換をすることが時々ある。

➡ 自分が看護師Aさんなら医師Cさんにどう伝える？

アサーティブなコミュニケーションのコツ

伝えるときのポイント

✓的をしぼる

要求の的をしぼり、１つの明確な要求を具体的に伝えましょう。

✓くりかえし自分のポイントに立ち戻る

落ち着いて自分の要求に立ち戻り、話がそれるのを避けましょう。

✓気持ちを言葉にする

その場や事実に対する自分の気持ちを感じたまま言葉にしましょう。

✓相手と自分の両方を尊重しよう

相手と自分、両方の要求と問題点をはっきりさせ、お互いの考えを尊重しましょう。

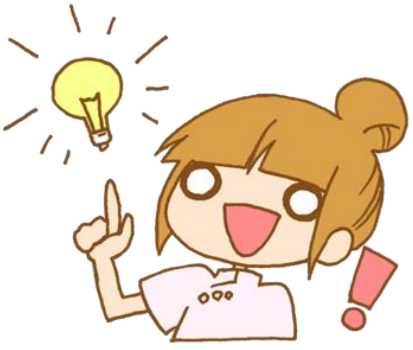

「み・かん・てい・いな」

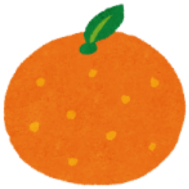

「み」	**見たこと・聞いたこと**	（客観的な）事実・状況を確認しよう
「かん」	**感じたこと**	（主観的な）考えや気持ちを含めよう
「てい」	**提案する**	的を絞って具体的に伝えよう
「いな」	**否と言われたら（断られたら）**	代案・折衷案を用意しておこう

- 自分の言いたいことを**「み・かん・てい・いな」**に当てはめて伝えるとアサーティブな表現に近づくことができます。

- 相手に「No（否）」と言われることはもちろんあります。あらかじめ、いくつかの代わりの案を考えておくと良いかもしれません。

（秋山剛・大野裕監修「うつ病の集団認知行動療法」 医学映像教育センター、2008）

さっきの例を「みかんていいな」にあてはめると…

患者の食事介助中に医師から要件を頼まれた看護師Aさん

看護師Aさんが配膳をしていると、ここ数日食欲がなくほとんど食事を食べていなかった患者Bさんが「今なら食べられそう」と言う。
看護師Aさんは「食欲を感じているこの機会を逃したくない」と思い、急いで配膳を済ませて食事介助を始めた。

すると、突然部屋に医師のCさんがやってきて「（他の患者さんの）包帯交換をするからすぐに来て」と言われた。医師のCさんは手術件数が多く忙しいため、昼休みの時間を使って包帯交換をすることが時々ある。

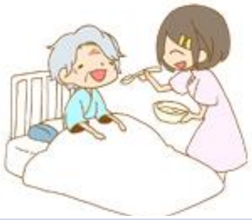

（例）

み	見たこと・聞いたこと<br>：客観的な事実・状況	ここ数日食欲がなくてなかなか食事が摂れなかった患者さんが、今なら食べられそうと仰っています。
かん	感じたこと<br>：主観的な考え・気持ち	私としてはこのタイミングを逃したくないと思っています。
てい	提案する<br>：具体的に伝える	食事介助が終わるまでお待ちいただけませんか？
いな	否と言われたら<br>：代案・折衷案を用意	それでは他の手の空いた看護師の方にお願いしていただけないでしょうか？

「みかんていいな」を使ってみよう

あなたは肺炎で入院中の患者Bさんを受け持ちました。Bさんを受け持ちをはじめて3日目。Bさんの肺炎は回復しましたが、ベッドで安静にしていた期間があったため筋力が低下しています。

いま必要な看護はADL（日常生活動作）を拡大することです。

この日、「洗面所まで行きましょう」と声をかけると、Bさんは「まだ体力も戻らないし、こんな年寄りにきついこと言わないで。おっくうだからタオルを持ってきて拭いてくれないかしら？」と言いました。

➡ 伝えたいことを「みかんていいな」に当てはめて考えてみましょう

み	見たこと・聞いたこと<br>：客観的な事実・状況	
かん	感じたこと<br>：主観的な考え・気持ち	
てい	提案する<br>：具体的に伝える	
いな	否と言われたら<br>：代案・折衷案を用意	

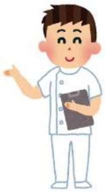

実際にやってみよう！

ロールプレイ：「頼む」

グループで取り組む場面を一つ選ぶ

□ **場面１：** B看護師が来年度から別の科へ移動となり、私が幹事となって送別会を開くことになった。以前B看護師のプリセプターをしていたベテランのA看護師を送別会に誘い、恒例となっている送別の一言をいただきたいのだが、A看護師はいつも「用事あるから」と言い、職場以外での集まりには積極的ではない。どのように提案すればよいだろうか？

□ **場面２：** 検査当日の朝になって患者さんから「検査続きで疲れた。これ以上検査は受けたくない」と言われた。主治医からは「治療方針の決定には必要だから患者を説得するように」と言われ、検査室からは「早く決めてほしい」と催促され、身動きが取れなくなってしまった。患者さんにどのように提案すればよいだろうか？

□ **場面３：** 患者さんが自分の病状や今後の治療方針を主治医に聞きたがっている。「先生が最近私の所に来てくれないんです。さじをなげているのではないでしょうか？」と不安を強めていた。患者さんの気持ちを主治医に伝えたいと思うが、主治医はいつも怒りっぽく、別の件で「忙しいから余計な仕事を増やすな」と怒鳴られた経験もある。主治医に対してどのように提案すればいいだろうか？

「みかんていいな」を使って 伝えたいこと・伝え方をまとめる

み	見たこと・聞いたこと<br>：客観的な事実・状況	
かん	感じたこと<br>：主観的な考え・気持ち	
てい	提案する<br>：具体的に伝える	
いな	否と言われたら<br>：代案・折衷案を用意	

ロールプレイ

- 役割を決める（3～４人１グループ：**本人役・相手役・観察者**）
  ＊観察者が進行＆書記＆発表
- 本人役の「みかんていいな」を使ってロールプレイ！
  （原稿は見ない）
- ロールプレイを終えて、それぞれの立場からの意見・感想を話し合う
  どんな気持ちになったか？どんな印象を受けたか？権利の行使・尊重はできた？改善すべき点は？（2回目のロールプレイに向けて）➡必要に応じて「みかんていいな」も修正！
- 話し合いを踏まえてもう1度ロールプレイ！

本人役	相手役	観察者

アサーティブの誤解

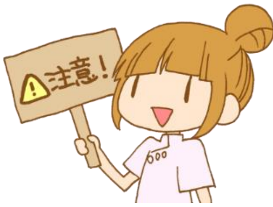

「アサーティブは人間関係における万能薬だ」

もちろん敬語や礼儀作法・タイミングも大切！
ボディーランゲージを適切に（言葉と表情の一致）

「これができればアサーティブ！という確固たる言動がある」

コツや方法を学んで、状況に合わせて使いこなそう！

「アサーティブに話をすれば相手は分かってくれる」

自分がアサーティブに表現したからといって相手が望み通りに反応してくれるわけではない。自分には自己表現する権利があるということを忘れないことが大事！

アサーティブの限界

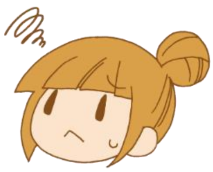

例：緊急入院が入ったので残業をした。時間外手当を申告するが師長から「悪いけど今月はこれ以上の時間外手当は予算オーバーでつけられないの」と言われた。「時間外手当をつけてほしい」と頼みたいが…

➡ 病院や看護部の看護師の労働環境を守るための仕組みが整っていないことが問題です。そのため、いくらアサーティブに師長に交渉しても進展しません。なぜならこの看護師を権利を脅かしているのは師長という個人ではなく、病院や看護部のシステムだからです。

例：外来の患者さんから「予約時間がとっくに過ぎている。いつも待たされてばかりだ」と苦情をいわれた。私のせいではないのに…と思いながらも、その場で対応しなければならないので困っている。

➡ 予約時間を毎回大幅に超過する外来のシステムに問題があります。患者さんは看護師に対して不満を言っているわけではありません。もちろん、患者さんの不満に対してアサーティブ・スキルを使うことはできますが、外来のシステムを変えない限り、同じ問題は何度も繰り返されます。

アサーティブのスキルで解決できる範囲と組織的に解決すべき問題とを見極めることも大切です！

第1部のまとめ

- ## 「アサーティブ」とは

「相手を尊重しつつ自分の権利も尊重し、誠実に、率直に、対等に自己表現すること」がアサーティブです。ポイントは相手を変化させることが目的ではない、ということ。コミュニケーションには大きく分けて３つのモード（いかり・ひるみ・いやみ）があり、そのときの自分のモードを知ることが大切です。

- ## アサーティブである権利

感じたこと・考えたことを表現してよいこと、人間は不完全であること、人は違っていて良いこと、アサーティブでいなくてもよいこと、という権利があることを忘れないようにしましょう。

- ## アサーティブなコミュニケーションのコツ

自分の言いたいことを「み・かん・てい・いな」に当てはめて伝えるとアサーティブな表現に近づくことができます。相手に「No（否）」と言われることはもちろんあります。あらかじめ、いつくかの代わりの案を考えておくと良いでしょう。

- ## 伝えるときのポイント

的をしぼる、くりかえし自分のポイントに立ち戻る、気持ちを言葉にする、ボディーランゲージを明確に、相手と自分の両方を尊重しよう

次回までのアクションプラン

- 今回のプログラムを踏まえて、今までの自分のコミュニケーションを振り返ろう。

- 次回までに「頼む」ことをアサーティブにやってみよう（最低１回）。

＊第2部の最初に振り返りをやります

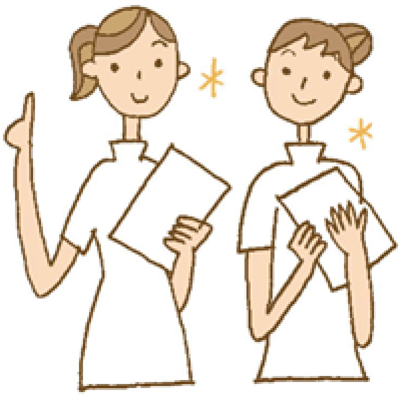

柔軟な考え方を身につけよう

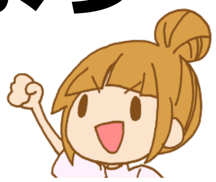

アサーティブになるために必要な３要素

ものごとの見方や考え方を柔軟にする

- 認知を柔軟にすることで、ネガティブな気持ちや行動を減らすことができます。

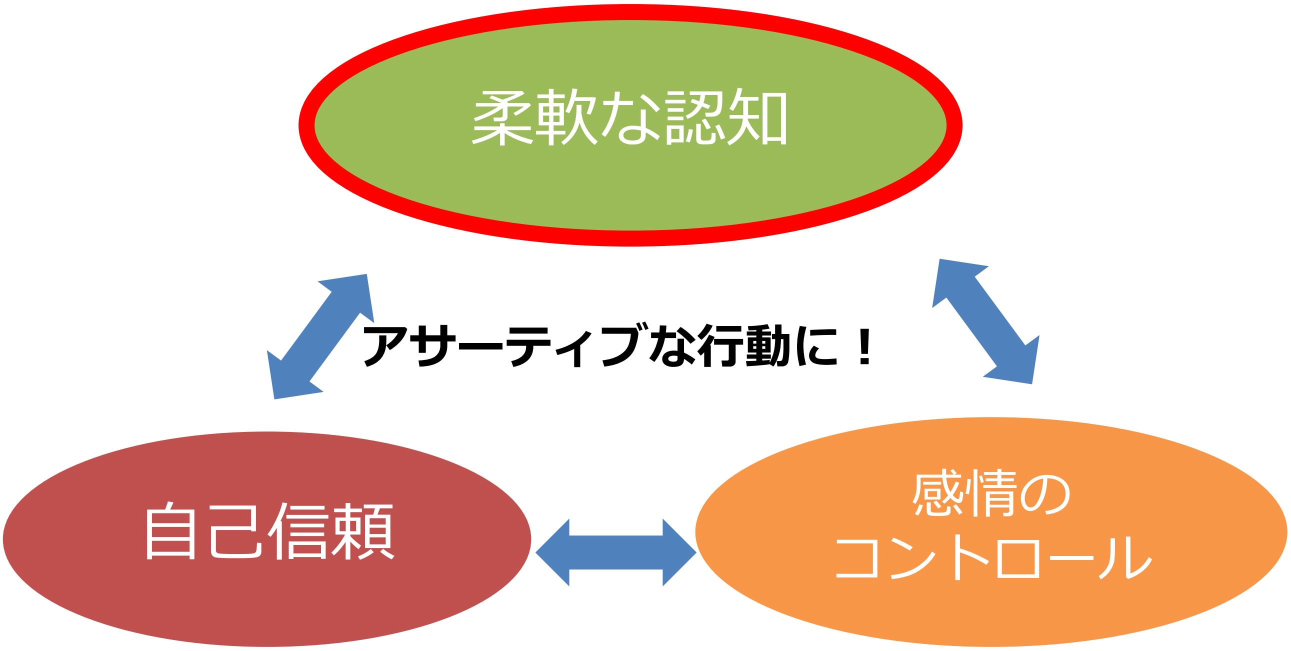

ありのままの自分を受け入れる

- 完璧な人間はいません。自信がない自分や失敗してしまう自分も受け入れることが大切です。

感じたことは、率直に、正直に表現してよい

- 怒りの感情であっても、相手を責めずに、率直に・正直に言葉にすることは大切です。すると相手も、こちらの真剣な気持ちに応えて、耳を傾けてくれるものです。

考え方のクセ

認知の歪み（Cognitive distortion）

A. 先読み

まだ起きていない出来事について「○○かもしれない」「○○にちがいない」と悲観的な予測を立ててしまう考え方

例）どうせ病棟の他の看護師さんと仲良くなれないに違いない

B. べき思考

「○○すべきだ」「○○しなければならない」と必要以上に思い悩んでしまいやすい考え方

例）自己犠牲を払ってでも、優しく共感的であるべきだ

考え方のクセ

C. 思い込み・レッテル貼り

自分が着目していることだけに目を向けて、根拠が不十分なのにも関わらず「いつも○○だ」「必ず○○だ」などと考え、「自分はダメ人間」などとレッテルを貼る。

例）ヒヤリ・ハットを起してしまい、「いつも失敗ばかり」「自分はダメ看護師」と考える。

D. 深読み

相手の気持ちを一方的に推測して、「きっとあの人は○○と考えているに違いない」と相手の心を読んでしまうような考え方。

例）患者さんに声をかけたときに「今はそっとしておいて」と言われ、「きっと自分のことを嫌っているのだろう」と考える。

考え方のクセ

E. 自己批判

良くないことが起きると、自分が原因と考えて、自分を強く責めてしまう考え方。

例）患者さんから病棟スタッフへの苦情があったと聞いて、「全部師長である私のせいだ」と考えて、必要以上に自分を責める

F. 白黒思考

灰色（あいまいな状態）に耐えられず、ものごとを白か黒か、良いか悪いかなどと極端にとらえてしまう考え方

例）「患者からの質問には何でも答えられなければならない」
「答えられないと専門職として失格だ」

なぜ柔軟な考え方が必要か

＜事実＞

一緒に処置をしていた後輩看護師に「そういうやり方もあるんですね～」と言われた

＜偏った考え方＞

先輩看護師として、後輩の前で間違いを犯してはならない。

- 「何か間違ったに違いない」
- 「頼りない先輩だとまた思われただろう」
- 「皮肉を言われて情けない」

＜結果＞

＜感情＞
- 落ち込む、憂うつ、悲しい、怖い、怒り

＜行動＞
- できるだけ個配と処置に入らないようにする
- **後輩に間違いがあっても指摘・指導しない**

＜柔軟な考え方＞

先輩看護師でも間違いはあるし、分からないことは教えてもらえばいい。

- 「気になること、知らないことがあったのかな」
- 「間違っていたなら教えてもらおう」
- 「もし知らないことがあるなら教えてあげよう」

＜結果＞

＜感情＞
- 恥ずかしい、楽しい

＜行動＞
- 自分が間違っていたか聞く
- 分からない、知らないことがあったのか聞く
- 自分の間違いがなかったか聞くし、後輩が知らないことがあれば教える

考え方次第でその後の気分・感情や行動が変わります

認知再構成法とは？

ある状況に対する自分の考え方・受け止め方が、自分の感情や行動をネガティブにしてしまうことがあります。認知再構成法は、そのような考えに対処する方法、いわば「**こころのストレッチ**」です。

具体的には、コラム表などを使って、そのときの客観的な状況・事実を捉え、自分を苦しくさせている認知（考え方や受け止め方）に気づき、バランスの取れた考え方を身につける練習をしていきます。

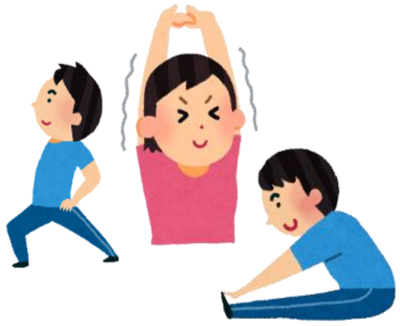

コラム表

**状況**<br>いつ・どこで・誰と・何を・どのようにしたかイメージできるように具体的なレベルで書く	いつ・どこで・誰と・何を・どのようにしたかイメージできるように具体的なレベルで書く
**そのときの気分・感情**<br>そのときの気持ち・感情を一語で表す<br>それぞれの感情の強さを（%）で表す	そのときの気持ち・感情を一語で表す（気分・感情の例を参照）<br>それぞれの感情の強さを（%）で表す
**そのときの考え**<br>そのとき頭に浮かんだ考えを書きだす<br>それぞれの考えに対する確信度（%）をつける<br>ネガティブな感情に結びつく一番重要な考えに○をつける	そのとき頭に浮かんだ考えを書きだす<br>ネガティブな感情に結びつく一番重要な考えに○をつける
**理由（根拠）**<br>そう考える理由を書きだす<br>客観的な事実だけ（予想は含まない）を言いきりの形で書く	そう考える理由<br>客観的な事実だけ（予想は含まない）を言いきりの形で書く
**反証**<br>そのときの考えを跳ね返す事実・反対の事実を書きだす（たくさんあるほど良い）	そのときの考えを跳ね返す事実・反対の事実を書きだす（たくさんあるほど良い）
**いまの考え**<br>理由と反証の両方を取り入れた文章で導き出したバランスのとれた考え方を書く（「理由」と「反証」を、「しかし」でつなぐ）	理由と反証の両方を取り入れた文章で導き出したバランスのとれた考え方を書く（「理由」と「反証」を、「しかし」でつなぐ）
**いまの気分、感情**<br>今の感情の強さを（%）で表す	気分の変化（%）

◆気分・感情の例

不安	憂うつ	悲しい	怖い
つらい	恥	がっかり	あきらめ
罪悪感	面白い	不満	パニック
楽しい	いらいら	落ち込み	うんざり
安心	爽快	うれしい	心配
絶望感	びっくり	やるせない	さわやか
誇らしい	愛情	屈辱感	傷ついた

気分・感情は０％か１００％（あり・なし）ではなく、様々な程度があります。

0　10　20　30　40　50　60　70　80　90　100　（%）

まったく　少し　中くらい　かなり　最大

◆反証のコツ

思い込み・レッテル貼り<br>「自分は○○な人間だ」	根拠と反証を見直しましょう。<br>可能性と解決策を具体的に考えましょう。<br>否定的に考えると失敗しやすくなります。
白黒思考	点数をつけるなど、段階的に考えましょう。<br>「必ず」「いつも」「絶対」は要注意。
べき思考	具体的な結果に目を向けて<br>解決策を考えてください。
自己批判	自分の長所・短所を<br>客観的に考えてみましょう。
先読み<br>深読み	反対の事実はないか<br>具体的に考えてみましょう。

コラム表：事例

**状況**<br>いつ・どこで・誰と・何を・どのようにしたかイメージできるように具体的なレベルで書く	今日の午前10時頃、病棟で一緒に処置をしていた後輩看護師に「そういうやり方もあるんですね～」と言われた。
**そのときの気分・感情**<br>そのときの気持ち・感情を一語で表す<br>それぞれの感情の強さを（%）で表す	落ち込む　60%　　憂うつ　85%<br>悲しい　50%　　怖い　45%
**そのときの考え**<br>そのとき頭に浮かんだ考えを書きだす<br>それぞれの考えに対する確信度（%）をつける<br>ネガティブな感情に結びつく一番重要な考えに○をつける	落ち込む　「皮肉を言われて情けない」<br>憂うつ　「頼りない先輩だとまた思われただろう」<br>悲しい　「後輩になめられてるんじゃないだろうか」<br>怖い　「何か間違ったに違いない」
**理由（根拠）**<br>そう考える理由を書きだす<br>客観的な事実だけ（予想は含まない）を言いきりの形で書く	• 以前、別の後輩と処置に入っていたときにマニュアルのやり方と違うことを指摘されたことがある。<br>• 後輩に知らないことを質問されて答えられなかったことがある。
**反証**<br>そのときの考えを跳ね返す事実・反対の事実を書きだす（たくさんあるほど良い）	• ベテランの看護師が処置を間違えていたことがある。<br>• 聞かれた質問の内の10個中8個は答えられる。<br>• 質問に答えられなかった後も、後輩は別の質問を自分にしてきた。
**いまの考え**<br>理由と反証の両方を取り入れた文章で導き出したバランスのとれた考え方を書く（「理由」と「反証」を、「しかし」でつなぐ）	• 処置を間違っていると指摘されたことがあり、今回も間違っている可能性が０ではないが、ベテラン看護師でも間違いはある。もし間違っていたとしても、また他の質問もしてくれたし、信頼がなくなるとは限らない。<br>• 確かに後輩の質問に答えられない時があるが、私以外の人に聞いてもらっていいし、私が知っていることは教えられる。<br>• 知らないことを「知らない」ときちんと言える方が誠実だ。
**いまの気分、感情**<br>今の感情の強さを（%）で表す	落ち込む　35%　　憂うつ　45%<br>悲しい　20%　　怖い　20%<br>恥ずかしい　30%　　わくわく　65%<br>安心　60%

●演習●
コラム表を完成させてみよう！
（一緒に1つずつ埋めていきましょう）

**状況**<br>いつ・どこで・誰と・何を・どのようにしたかイメージできるように具体的なレベルで書く	明日の午後１３時頃から、病棟で自分が受け持っている患者さんについてのカンファレンスがある。司会者は、普段から新人や後輩に対して怒鳴りつけている苦手な先輩看護師。
**そのときの気分・感情**<br>そのときの気持ち・感情を一語で表す<br>それぞれの感情の強さを（%）で表す	
**そのときの考え**<br>そのとき頭に浮かんだ考えを書きだす<br>それぞれの考えに対する確信度（%）をつける<br>ネガティブな感情に結びつく一番重要な考えに○をつける	
**理由（根拠）**<br>そう考える理由を書きだす<br>客観的な事実だけ（予想は含まない）を言いきりの形で書く	
**反証**<br>そのときの考えを跳ね返す事実・反対の事実を書きだす（たくさんあるほど良い）	
**いまの考え**<br>理由と反証の両方を取り入れた文章で導き出したバランスのとれた考え方を書く（「理由」と「反証」を、「しかし」でつなぐ）	
**いまの気分、感情**<br>今の感情の強さを（%）で表す	

実際にやってみよう！

ロールプレイ：「断る」

グループで取り組む場面を一つ選ぶ

□ **場面１：**先輩の看護師から院内看護研究を一緒にしないか、と誘われた。興味があるが、今は院内の仕事をたくさん抱えているので断りたい。どのようにお断りすればよいだろうか？

□ **場面２：**マッサージを好む患者。「もういいよ」と言うまで１時間かかる時もあり、他の仕事へ少なからず支障が出始めていた。今日こそは15分程度で切り上げるためにお断りを入れようと思う。どのようにお断りすればよいだろうか？

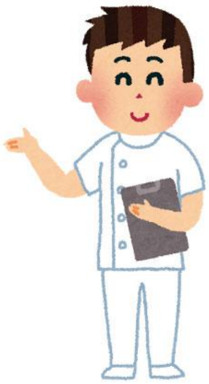

どんな認知が邪魔してきそうか？

□ **場面１：**先輩の看護師から院内看護研究を一緒にしないか、と誘われた。興味があるが、今は院内の仕事をたくさん抱えているので断りたい。どのようにお断りすればよいだろうか？

➡

□ **場面２：**マッサージを好む患者。「もういいよ」と言うまで１時間かかる時もあり、他の仕事へ少なからず支障が出始めていた。今日こそは15分程度で切り上げるためにお断りを入れようと思う。どのようにお断りすればよいだろうか？

➡

アサーティブな心構えで取り組もう！

□ **場面１：**先輩の看護師から院内看護研究を一緒にしないか、と誘われた。興味があるが、今は院内の仕事をたくさん抱えているので断りたい。どのようにお断りすればよいだろうか？

□ **場面２：**マッサージを好む患者。「もういいよ」と言うまで１時間かかる時もあり、他の仕事へ少なからず支障が出始めていた。今日こそは15分程度で切り上げるためにお断りを入れようと思う。どのようにお断りすればよいだろうか？

選んだ場面で…

どんな認知が邪魔しそうか？

どんな認知（適応的な考え方）だと行動に移れそうか？

と考える

「みかんていいな」を使って 伝えたいこと・伝え方をまとめる

み	見たこと・聞いたこと<br>：客観的な事実・状況	
かん	感じたこと<br>：主観的な考え・気持ち	
てい	提案する<br>：具体的に伝える	
いな	否と言われたら<br>：代案・折衷案を用意	

ロールプレイ

- 役割を決める（3～４人１グループ：**本人役・相手役・観察者**）
  ＊観察者が進行＆書記＆発表
- 本人役の「みかんていいな」を使ってロールプレイ！
  （原稿は見ない）
- ロールプレイを終えて、それぞれの立場からの意見・感想を話し合う
  どんな気持ちになったか？どんな印象を受けたか？権利の行使・尊重はできた？改善すべき点は？（2回目のロールプレイに向けて）➡必要に応じて「みかんていいな」も修正！
- 話し合いを踏まえてもう1度ロールプレイ！

本人役	相手役	観察者

ほめるときのコツ

• 具体的な行動をほめる

「さっきのやつよかったよ～。いや～すごいね～。」

↓

「さっきのミーティングでは、**テキパキ議事の進行をして、皆が発言できるように工夫してくれて**本当によかった。とてもいいミーティングになったよ。ありがとう。」

どういった点がどのように良かったのかを伝える

過剰なプラス評価ではなく正当な評価を！

• 他の人と比較しない（絶対的評価）

「あの人は仕事が遅いのに、あなたは仕事が早いわね（相対的評価）」

↓

「あなたは手際もよくて、テキパキとよく動いてくれて本当に頼もしいです。」

ほめるときには本人のことを話題にする

• 期間をあけず、タイムリーにほめる

本人がはっきり思い出せないようなタイミングでは、あまり効果が期待できません。

• 余計な言葉を付け加えない

「今日のカンファレンスでの新患紹介は良かったと。でも、もっと大きな声でハキハキ話さないと。」

↓

「今日のカンファレンスでの新患紹介は、事前の情報収集がしっかりされていて良かったよ。患者さんが何に困っているか皆に伝わったと思うよ。」

（とくに立場が上の人は）ほめた後に、ついつい余計な言葉（小言）を付け加えてしまいがちです。気をつけましょう。

ほめた後の「でも」に注意

ほめられるときのコツ

- **「照れるな～」「恥ずかしいな・・・」と言ってみる**
  （気持ちの開示）

- **「そう言ってもらえてうれしい。ありがとう」と言う**
  （相手の「ほめ」を受け止める：素直な同意）

- **いいところは具体的に聞いてもよい**
  「どんなところがお役に立てましたか？」

謙虚さは大切だが、
相手の「ほめたい」という気持ちを
素直に受け入れよう。

「謙虚」とは控えめ・つつましい・**素直**という意味

例：ほめる

場面：精神科病棟に勤務している看護師長。ある日、うつ病の患者さんが勤務４か月の新人看護師Bの対応に、「以前はつらいだけでしたがあの若い看護師さんがとても親切で、私の話をじっくり聞いてくれるので病棟での毎日が楽しいです。退院に向けて前向きになれます。」と語った。新人看護師Bにあなたの賞賛も含めてこのことを伝えてみよう。

み	見たこと・聞いたこと<br>：客観的な事実・状況	○○さんが「あなたが話を丁寧に聞いてくれるから病棟での生活が充実して、退院に前向きになれる」と言ってたわよ。
かん	感じたこと<br>：主観的な考え・気持ち	私にわざわざ言いに来られたということは、本当に感謝されていたんだと思うの。業務に追われる中で患者さんとしっかり向き合うことはなかなか難しいことよ。私も病棟の管理者として嬉しいわ。これからも続けていってね。
てい	提案する<br>：具体的に伝える	
いな	否と言われたら<br>：代案・折衷案を用意	

例：ほめられる

場面２：産婦人科、外科の混合病棟に異動してきたばかりの看護師。ある勤務の時、師長から「あなたと勤務だと色んな部署で経験をしてるから頼もしいし心強いわ。」と言われた。師長にどのように反応を返せばよいでしょう？

み	見たこと・聞いたこと<br>：客観的な事実・状況	ここの部署に来て日が浅いので、皆さんに教えて頂くことの方が多いのですが
かん	感じたこと<br>：主観的な考え・気持ち	そう言ってもらえると大変嬉しいです。励みになります。ありがとうございます。
てい	提案する<br>：具体的に伝える	
いな	否と言われたら<br>：代案・折衷案を用意	

自分に最近あった場面を選ぶ

- 自分が最近経験した「ほめる」「ほめられる」を思い出してください。
- これからほめようと思っていること、ほめて欲しいことでも構いません。
- 「もっと上手に、ほめることができた（ほめられた）かな～」と思う場面だとやりやすいでしょう。

- 職場での出来事でもいいですし、日常生活の出来事でも構いません。

例：部下が良い仕事をした、先輩の発表が素晴らしかった、患者さんが食事制限と運動を頑張っていた、仕事が早いとほめられた、情報収集のために早く病棟に来ていること（ほめて欲しいこと）、夫が家事を手伝ってくれた、バーで隣に座った子が可愛かった

あなたが選んだ場面（誰に対して、何をほめる・ほめられる）

「みかんていいな」を使って
伝えたいこと・伝え方をまとめる

み	見たこと・聞いたこと<br>：客観的な事実・状況	
かん	感じたこと<br>：主観的な考え・気持ち	
てい	提案する<br>：具体的に伝える	
いな	否と言われたら<br>：代案・折衷案を用意	

ロールプレイ

- 役割を決める（3～４人１グループ：**本人役・相手役・観察者**）
  ＊観察者が進行＆書記＆発表
- 本人役の「みかんていいな」を使ってロールプレイ！
  （原稿は見ない）
- ロールプレイを終えて、それぞれの立場からの意見・感想を話し合う
  どんな気持ちになったか？どんな印象を受けたか？権利の行使・尊重はできた？改善すべき点は？（2回目のロールプレイに向けて）➡必要に応じて「みかんていいな」も修正！
- 話し合いを踏まえてもう1度ロールプレイ！

本人役	相手役	観察者

第2部のまとめ

- **柔軟な考え方を身につけよう：**アサーティブになるために「認知再構成法」のコラム表を用いて、ものの見方や考え方を柔軟にしましょう。客観的な状況・事実を捉えることで、自分を苦しくさせている認知（考え方や受け止め方）に気づき、バランスの取れた考え方を身につけることができます。

- **ロールプレイ「断る」：**相手の要求や頼みを断ろうとする時に出てくる認知に気づき、認知再構成法などを用いて考え方を柔軟（アサーティブな心構え）にすることがコツです（自分が提案するときにも使えます）。なにがなんでも相手の要求を断ろうとするのではなく、自分にとっても相手にとっても受け入れやすい断り方、場合によっては代案・折衷案を考えることが大切です。

- **ロールプレイ「ほめる・ほめられる」：**ほめるときは、具体的にほめること、そして他人と比較しないことがコツです。ほめられるときは、自分の気持ちの開示すること、相手の気持ちを素直に受け入れること、そして、いいところを具体的に聞くことがコツです。

今後の課題・目標のイメージ

・6ヶ月後までの目標・課題

アサーティブなコミュニケーションを身につける上での課題・目標をイメージして書き出してみよう（複数可）！

例）仕事をお願いするときに、遠慮して要求の全てを伝えられないので、やってほしいところまでしっかり伝える。

学び・今後の課題を共有しよう！

学び、感想

トレーニングを通して学んだことや感想を共有しよう！

課題・目標の共有

書きだした6か月後までの課題・目標を発表しよう！

一人1分ずつで簡単にお話しください。

研修後のアクションプラン

- 「頼む」「断る」「ほめる・ほめられる」について、最低一回ずつ取り組んでみましょう！

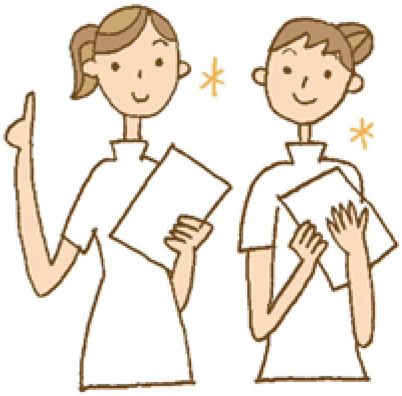

引用・参考文献

- 平木典子、沢崎達夫、野末聖香「ナースのためのアサーション」金子書房（2002/12）
- 森田汐生「心が軽くなる！気持ちのいい伝え方」株式会社主婦の友社（2015/11）
- 森田汐生「もっとことばに出そう！自分の気持ち」株式会社すばる舎（2004/11）
- 勝原裕美子「Beアサーティブ！現場に活かすトレーニングの実際」医学書院 (2003/07)
- 大野裕「こころのスキルアップ・プログラム：認知療法・認知行動療法の視点から」国立精神・神経医療センター認知行動療法センター（URL：http://www.cbtjp.net/）

＜プログラム制作者＞

宮崎大学大学院看護学研究科	中村陽平
宮崎大学テニュアトラック推進機構	吉永尚紀
宮崎大学医学部看護学科	田上博喜

＜二次利用について＞

本資料の全部または一部を、無断で複製・複写（コピー）すること、第三者に譲渡及びセミナー等の資料・テキストとして使用することは、著作権法上での例外（学業・個人での利用）を除き禁じられています。複写、使用する場合は制作者の許諾を得てください。

2016年　3月　　第1版

2016年　5月　　第2版

アサーティブ・トレーニング

解答例

目次

＜第１部＞

- 「みかんていいな」を使ってみよう　………2
- 上手に頼む（ロールプレイ①）　………3
  場面1、場面2、場面3

＜第２部＞

- コラム表を完成させてみよう！　………5
- 上手に断る（ロールプレイ②）　………6
  場面1、場面2

「みかんていいな」を使ってみよう

（例）

み	見たこと・聞いたこと<br>：客観的な事実・状況	ずっと長い時間ベッドで横になっていたので、体力が落ちておっくうになりますよね。
かん	感じたこと<br>：主観的な考え・気持ち	そのような時に動くのは本当に大変だと思いますが、今動かないと体力の回復がどんどん遅れてしまうので私は心配です。Bさんに早く元気になってほしいと思っています。
てい	提案する<br>：具体的に伝える	ゆっくりで良いので、洗面所まで歩いてみませんか？
いな	否と言われたら<br>：代案・折衷案を用意	私が付き添いますので、一緒に歩いていきませんか？

例：「頼む」 場面１

み	見たこと・聞いたこと<br>：客観的な事実・状況	今年度でＢさんが別の科へ移動になったため、送別会を開くことになりました。
かん	感じたこと<br>：主観的な考え・気持ち	Ａさんはプリセプターをされていたので、Ａさんに参加していただけるとＢさんは喜ばれると思いますし、幹事の私としてもありがたいです。
てい	提案する<br>：具体的に伝える	ぜひ送別会にご参加いただけませんか？また、そのときに（恒例となっている）元プリセプターからの送別メッセージも頂きたいと思っています。
いな	否と言われたら<br>：代案・折衷案を用意	それでは、メッセージを手紙のような形で書いて頂き、私が代読するというのはいかがでしょうか？

例：「頼む」 場面２

み	見たこと・聞いたこと<br>：客観的な事実・状況	これ以上は検査を受けたくないと思われるほど、検査続きで疲れていらっしゃるんですね。
かん	感じたこと<br>：主観的な考え・気持ち	○○さんが非常に疲れていらっしゃるご様子なので私も休んでいただきたいのですが、検査が長引くことで○○さんの治療が遅れてしまうことが心配です。
てい	提案する<br>：具体的に伝える	お身体がキツイと思いますが、検査を早めに済まされてから、午後はお休みになるというのはいかがですか？
いな	否と言われたら<br>：代案・折衷案を用意	検査の順番を午後に回してもらって休憩時間をいただくので、少し休んだら午後から検査に向いませんか？

例：「頼む」　場面３

み	見たこと・聞いたこと<br>：客観的な事実・状況	先生、〇〇さんが現在のご自分の病状やこれからの治療方針をお知りになりたいご様子です。とても不安な気持ちを口にされています。
かん	感じたこと<br>：主観的な考え・気持ち	お忙しいとは思いますが、一度〇〇さんと現在の状況や今後のことをお話しると、〇〇さんも安心されるのではないかと思います。
てい	提案する<br>：具体的に伝える	〇〇さんとお時間をとってお話しされてみてはいかがでしょうか？
いな	否と言われたら<br>：代案・折衷案を用意	朝の回診のときに「心配なことや不安なことはないですか？」とお声かけいただけないでしょうか？

●演習●
コラム表を完成させてみよう！

**状況**<br>いつ・どこで・誰と・何を・どのようにしたかイメージできるように具体的なレベルで書く	明日の午後１３時頃から、病棟で自分が受け持っている患者さんについてのカンファレンスがある。司会者は、普段から新人や後輩に対して怒鳴りつけている苦手な先輩看護師。
**そのときの気分・感情**<br>そのときの気持ち・感情を一語で表す<br>それぞれの感情の強さを（%）で表す	怖い　80%　　不安　70%　　憂うつ　45%
**そのときの考え**<br>そのとき頭に浮かんだ考えを書きだす<br>それぞれの考えに対する確信度（%）をつける<br>ネガティブな感情に結びつく一番重要な考えに○をつける	怖い　「的外れなことや、間違ったことを言ったら先輩に怒られる」<br>不安　「その場が重い雰囲気になってしまう」<br>憂うつ　「いつもくよくよ悩んでしまって情けない」
**理由（根拠）**<br>そう考える理由を書きだす<br>客観的な事実だけ（予想は含まない）を言いきりの形で書く	• 先輩に「情報収集が足りないんじゃないの」と怒鳴られたことがある。<br>• 先輩が他の看護師にカンファレンスでも怒鳴りつけていたことがある。
**反証**<br>そのときの考えを跳ね返す事実・反対の事実を書きだす（たくさんあるほど良い）	• 先輩が怒鳴らなかった日もある。<br>• 先輩が師長から「怒鳴っている内容自体が的外れなことがあり、師長に注意されていた。<br>• 病棟の7割は先輩のことが嫌いと言っていた。<br>• カンファレンスは、問題点や課題を改善・解決していくためのもの。<br>• この患者さんと話しをしている時間が一番長いのは受持ち看護師の私。
**いまの考え**<br>理由と反証の両方を取り入れた文章で導き出したバランスのとれた考え方を書く（「理由」と「反証」を、「しかし」でつなぐ）	• 確かに、その先輩は怒りっぽいし、その場が凍り付く可能性はある。<br>• しかし、その患者さんのことを一番知っているのは私だし、そこで意見を言わないのはむしろ無責任。<br>• 万が一間違ったことをいったとしても、「カンファレンスでは間違いを正す」ことが目的なので問題ないし、改善・解決策をみんなで話し合えばいい。それが一番患者のためになるし、私もくよくよ悩まなくて済む。
**いまの気分、感情**<br>今の感情の強さを（%）で表す	怖い　35%　　不安　40%　　憂うつ　20%　　安心　30%

例：「断る」 場面１

み	見たこと・聞いたこと<br>：客観的な事実・状況	お誘いありがとうございます。ですが、今は病棟で任されている仕事が多く、ほとんど片付いていません。
かん	感じたこと<br>：主観的な考え・気持ち	研究に興味はあるのですが、今は病棟の仕事が片付いておらず、そちらも責任を持ってやりたいと考えているので、気持ちにも時間にも余裕がありません。
てい	提案する<br>：具体的に伝える	2か月後には院内の仕事が片付くので、それまでお待ちいただくことは可能ですか？
いな	否と言われたら<br>：代案・折衷案を用意	それでは私が現在持っている仕事を他の方に振り分けて頂くことはできませんか？

例：「断る」 場面２

み	見たこと・聞いたこと<br>：客観的な事実・状況	□□さん。以前１時間のマッサージをしていて、他の患者さんへのケアが遅れてしまったことがありました。
かん	感じたこと<br>：主観的な考え・気持ち	□□さんの要望にも出来る限りお応えしたいのですが、今日は他の患者さんへのケアもあり、そちらも平等に行いたいと考えています。
てい	提案する<br>：具体的に伝える	今日は15分程しかお時間が作れないのですが、それでもよろしいでしょうか。
いな	否と言われたら<br>：代案・折衷案を用意	それでは、今日の業務リーダーに他に手の空いたスタッフがいないか相談してきてもよろしいでしょうか？
